# Ketogenic Diet for a Young Adult Patient With Chronic-Phase Febrile Infection-Related Epilepsy Syndrome

**DOI:** 10.7759/cureus.22099

**Published:** 2022-02-10

**Authors:** Koji Obara, Tomoko Ono

**Affiliations:** 1 Neurology, National Hospital Organization Akita National Hospital, Yurihonjo, JPN

**Keywords:** refractory epilepsy, ketogenic diet, fires, febrile infection-related epilepsy syndrome, adult

## Abstract

Febrile infection-related epilepsy syndrome (FIRES) is a rare disease, whereby refractory status epilepticus (a severe epileptic syndrome) occurs in previously healthy individuals following a febrile illness. Here, we report a patient with FIRES who received ketogenic diet (KD) therapy initiated in the chronic phase. A 21-year-old man presented with status epilepticus, following fever and headache. In the acute phase, his seizures were refractory to conventional antiepileptic drugs and were suppressed only by intravenous anesthetics. In the chronic phase, he showed frequent seizures with concurrent severe cognitive decline. Twenty-seven months after onset, the patient was started on KD. Consequently, his seizure frequency rapidly reduced while his cognitive function slowly improved, albeit incompletely. Recently, KD has been shown to both reduce seizures and improve cognitive prognoses in children with FIRES. Although early KD may help in both seizure control and cognitive prognosis, it is likely that KD can be applied to adult patients with chronic FIRES.

## Introduction

Febrile infection-related epilepsy syndrome (FIRES) is an epileptic encephalopathy in which previously healthy individuals present with prolonged status epileptics (SE) following a febrile illness [1-3]. The SE is refractory to conventional antiepileptic drugs (AEDs) and immunotherapies [2-3]. Although seizure frequency gradually decreases in the chronic phase of FIRES, in many cases, these last throughout the patient’s lifetime and is accompanied by cognitive decline [2-3]. Recently, several small case studies report that the ketogenic diet (KD) both reduces seizures in FIRES and improves cognitive prognosis in children and in the acute phase of this disease, with incompletely understood mechanisms [4-5]. We report a young adult man with FIRES, who showed great seizure reduction and mild improvement of cognitive function with long-term KD despite delayed initiation in the chronic phase.

## Case presentation

**Figure 1:**
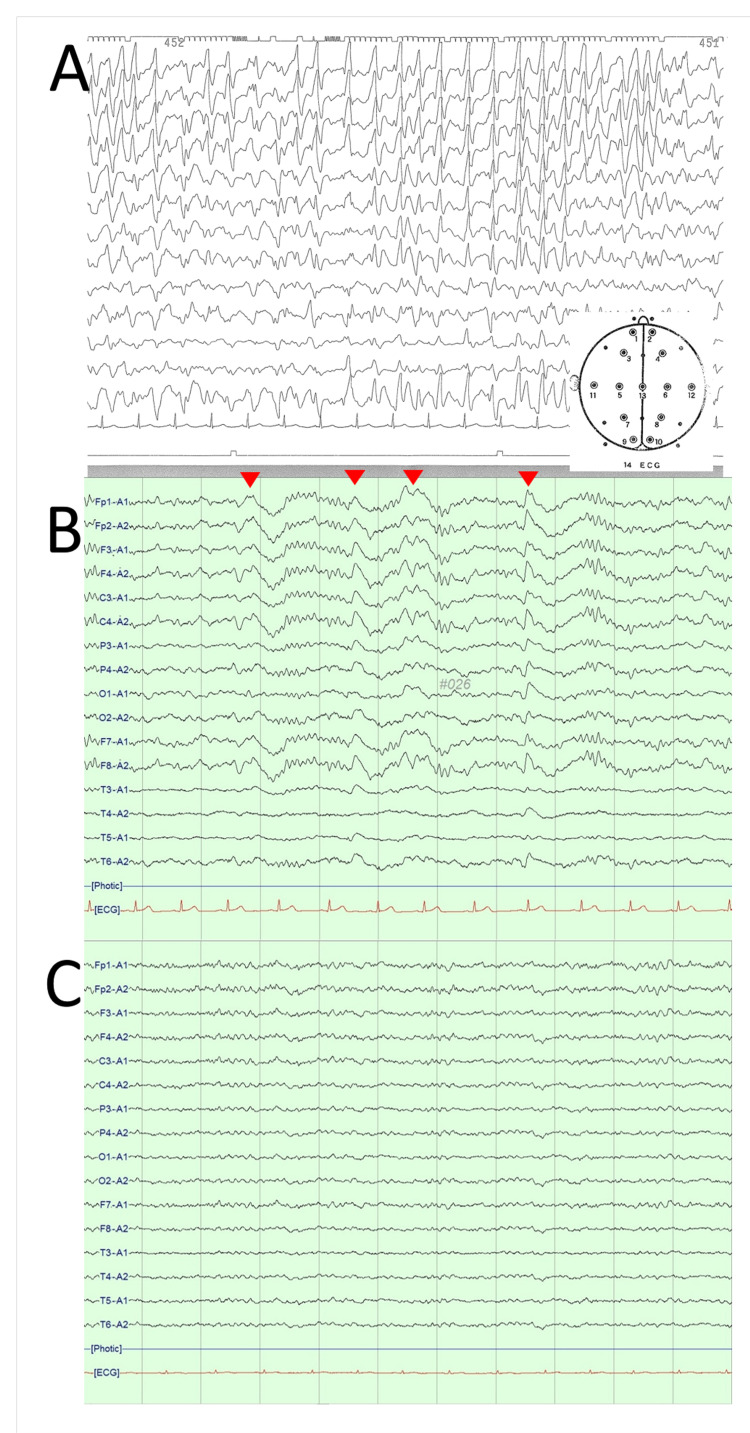
Electroencephalogram (EEG) finding (A) Soon after tapering intravenous thiamylal sodium at 20 days after the onset, EEG showed a burst of high-amplitude sharp waves in the predominantly frontoparietal region. (B) Fourteen months after the onset, interictal EEG shows repeated slow waves in the frontoparietal region (arrowheads). (C) Thirty-six months after the onset and nine months after ketogenic diet initiation, EEG shows no spikes, sharp waves, and slow waves.

A previously healthy, 21-year-old man presented with fever and headache. Three days later, he exhibited a disturbed consciousness level, frequent twitching of the ipsilateral face and upper limb, and apneic spells. Brain magnetic resonance imaging (MRI) showed no abnormalities. A cerebrospinal fluid (CSF) study disclosed slight lymphocytic pleocytosis with 8 leukocytes/mm^3^, including 75% lymphocytes and total protein 0.4 g/l (normal <0.5 g/l). No organism was isolated on Gram staining and culture of the CSF, and the polymerase chain reaction was negative for herpes simplex virus, cytomegalovirus, Epstein-Barr virus, varicella-zoster virus, and human herpesvirus 6. A CSF panel of autoimmune encephalitis, including anti-N-methyl-D-aspartate receptor, gamma-aminobutyric acid B receptor, leucine-rich glioma-inactivated 1, and contactin-associate protein 2 antibodies were all negative. As his seizures periodically repeated every five to 10 minutes, he was intubated and ventilated using a mechanical respirator and treated with midazolam. Five days after the onset, he was given fosphenytoin and levetiracetam sequentially in addition to midazolam, but his seizures were not controlled. Eight days after the onset, he was controlled with burst-suppression on electroencephalogram (EEG) using a continuous infusion of midazolam, propofol, and thiamylal sodium. However, the seizures and continuous epileptic discharge often recurred when these anesthetics were tapered (Figure 1A).

He was started on glucocorticoid pulse therapy, plasma exchange (PE), and high-dose intravenous immunoglobulin (IVIG) sequentially. Three months after onset, his frequency of seizures decreased under oral administration of six AEDs (levetiracetam 3,000 mg/day, lamotrigine 100 mg/day, perampanel 12 mg/day, topiramate 600 mg/day, phenobarbital 120 mg/day, and potassium bromide 2.25 g/day), and his intravenous anesthetics and artificial ventilator could be discontinued. There was persistent weakness in both lower limbs, probably due to critical illness neuropathy. Seven months after the onset, he was transferred to our hospital. At the same time, a physical examination revealed a temperature of 36.6 °C, a pulse of 82 beats/min, a blood pressure of 128/62 mmHg, a height of 163 cm, and a bodyweight of 62 kg. On neurological examination, he was alert but apathetic. Muscle weakness (2/5 on the Medical Research Council muscle grading scale) and moderate hypesthesia and areflexia were observed in bilateral lower limbs. Laboratory studies were unremarkable. At our hospital, he presented apneic spells and impaired consciousness 15-20 times a week and tonic-clonic seizures two to three times a month. AED adjustments did not reduce the frequency or duration. In addition to his lack of spontaneity, he became agitated and impulsive and showed behavioral problems such as sexual disinhibition and stereotyped tool use, which were resistant to all administered antipsychotic drugs. Fourteen months after the onset, interictal EEG showed repeated slow waves in the frontoparietal region (Figure 1B). Wechsler Adult Intelligence Scale-Fourth Edition (WAIS-IV) showed full-scale IQ 52. Wechsler Memory Scale-Revised (WMS-R) was less than 50 in all categories (Table 1).

**Table 1:** Neuropsychological tests

After ketogenic diet initiation (month)	Before	3	9	16
After the onset (month)	14	30	36	43
Wechsler Adult Intelligence Scale - Fourth Edition				
Full-scale IQ	52	55	61	67
Verbal comprehension index	67	68	68	81
Perceptual reasoning index	58	69	78	82
Working memory index	67	60	69	67
Processing speed index	54	57	57	57
Wechsler Memory Scale - Revised				
Verbal memory index		<50	<50	<50
Visual memory index		<50	76	90
General memory index		<50	<50	50
Attention and concentration index		<50	54	69
Delayed recall index		<50	<50	<50

**Figure 2:**
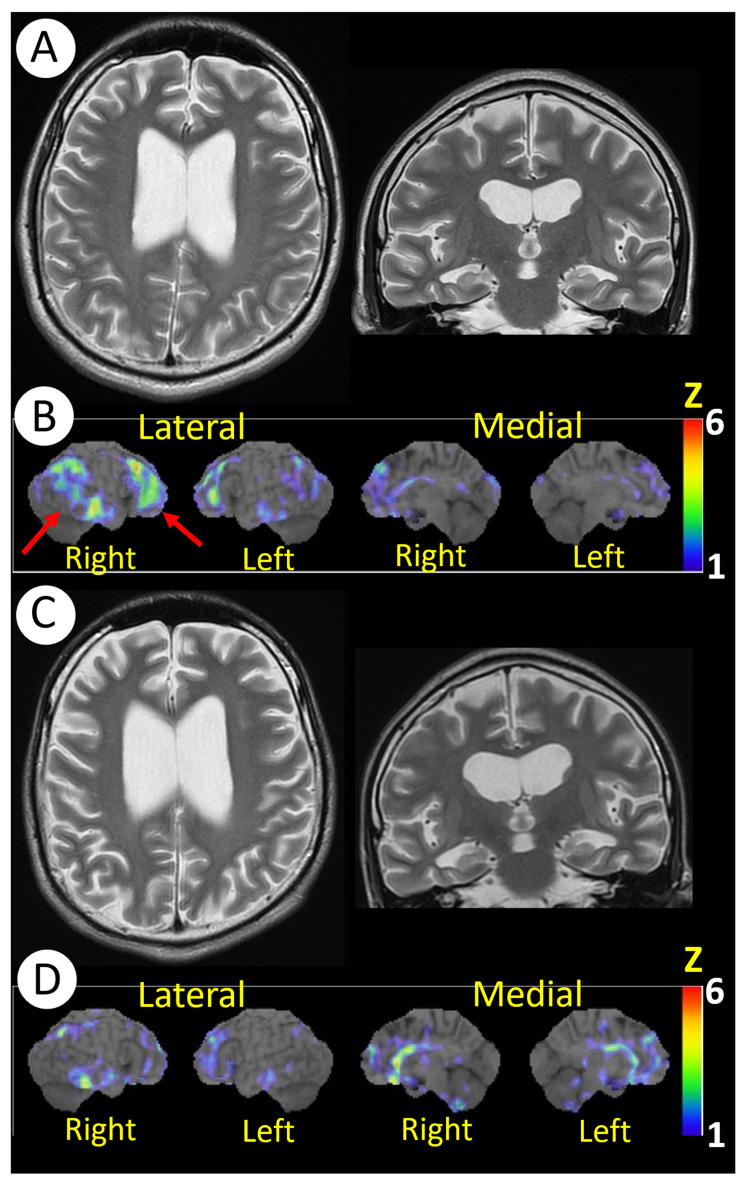
MRI and SPECT The findings of brain magnetic resonance imaging (MRI) and cerebral blood flow single-photon emission computed tomography using N-isopropyl-p-[^123^I] iodoamphetamine (SPECT). Twenty-seven (27) months after the onset: (A) MRI shows atrophy of the bilateral frontal lobes and mesial temporal lobes and dilatation of the lateral ventricles. (B) SPECT reveals hypoperfusion in the right-dominant frontal and parieto-temporal cortices (arrows). Thirty-six (36) months after the onset and nine months after the ketogenic diet initiation: (C) MRI shows no changes compared to (A) before the introduction of the ketogenic diet. (D) SPECT shows the improvement of hypoperfusion in the frontal and parieto-temporal cortices. The Z-score on SPECT is higher as the degree of decrease in cerebral blood flow is larger than that of an age-matched normal database.

Twenty-seven months after onset, brain magnetic resonance imaging (MRI) showed atrophy of the bilateral frontal lobes and mesial temporal lobes and dilatation of the lateral ventricles (Figure 2A).

Single-photon emission CT using N-isopropyl-p-[123I] iodoamphetamine (IMP-SPECT) revealed hypoperfusion in the right-dominant frontal and parieto-temporal cortices (Figure 2B). Then, we started the patient on a 2:1 (fat:carbohydrate and protein) ratio KD of 1,350 kcal. Within 48 hours of the KD initiation, he achieved a urine ketone of 3+. One month after KD initiation, laboratory tests showed elevated total ketone body level 2,792 umol/L, acetoacetic acid level 716 umol/L, and 3-hydroxybutyric acid level 2,076 umol/L. His lamotrigine and topiramate were safely discontinued. Subsequently, tapering of AEDs was attempted but was finally fixed with four AEDs (levetiracetam 2,000 mg/day, perampanel 8 mg/day, phenobarbital 120 mg/day, and potassium bromide 2.1 g/day) because further AED reduction increased apneic spells even under KD. At three-month follow-up after the KD initiation, his seizures had reduced >75%, his behavioral problems gradually subsided, and his attention and durability during rehabilitation improved. Thirty-four months after the onset and seven months after the KD initiation, he was discharged home. At 36 months after the onset, his weight decreased by 10 kg since KD initiation, and he was then administered a 1.5:1 ratio KD of 1,800 kcal. At the same time, his frequency of seizures was once or twice a week. He could voluntarily write a letter and draw a simple picture. EEG revealed no spikes, sharp waves, or abnormal slow waves (Figure 1C). Brain MRI showed no change with regard to cerebral atrophy (Figure 2C). IMP-SPECT showed improvement of hypoperfusion in the right-dominant frontal and parieto-temporal cortices (Figure 2D). Table 1 shows the results of neuropsychological tests during the clinical course. Finally, WAIS-IV revealed particular improvement in perceptual reasoning with full-scale IQ 67. WMS-R showed improvement in visual memory. However, processing speed and verbal memory did not improve, and he required significant support in most activities of daily living.

## Discussion

Febrile infection-related epilepsy syndrome (FIRES) is a rare disease in which refractory status epilepticus (severe epileptic syndrome) occurs in mostly previously healthy children and young adults following a febrile illness [1-3]. These seizures are very resistant to conventional AEDs and can be suppressed only with high-dose intravenous barbiturate administration [2-3]. Although some autoimmune inflammation is presumed as the pathomechanism of FIRES, conventional immunotherapies, including steroids, IVIG, and PE, are usually disappointing [2-3]. Recently, several small studies have reported that KD could both reduce seizures and improve intellectual prognosis in FIRES. These findings were mainly in children in the acute phase where continuous anticonvulsant infusion was required to suppress seizures [4-5].

Herein, we reported a young adult case with FIRES, who received KD in the chronic phase and long-term. Despite delayed KD initiation, he showed a great decrease in seizure frequency. However, the improvement in cognitive function was mild and insufficient.

The ketogenic diet (KD) is a high-fat, low-carbohydrate, and moderate protein content and has been used to treat children with refractory epilepsy since the 1920s, with a resurgence in the past two decades [6]. Putative mechanisms of KD include anti-inflammatory effects and neuroprotective properties in addition to anticonvulsant effects [7]. These various effects of KD may contribute to both seizure reduction and cognitive improvement in children with refractory epilepsies including FIRES [4-5].

Compared to children, the use of the KD in adolescents and adults has been more restrained due to reasons such as an inability to adhere to the diet or a medical team that is not proficient in administering the diet [8]. However, the emergence of more liberal regimens (e.g., the modified Atkin’s diet, the low-glycemic index treatment, and the medium-chain triglyceride diet) has allowed adolescents and adults to participate in diet therapies more tolerably, and these regimens may reduce seizures similar to classic KD with 4:1 ratio of fat to protein and carbohydrate [8]. Consequently, growing evidence indicates that the KD and its variants can be effective for adolescents and adults with refractory epilepsy as well as in children [8-11]. The seizure frequency of our patient was also sufficiently reduced on KD with lower ratios, such as 2:1 or 1.5:1, compared to the classic 4:1.

KD should be initiated as early as possible in order to achieve and sustain seizure control, particularly status epileptics in the acute phase of FIRES, which can lead to deleterious cognitive outcomes [5,8]. Moreover, it has been reported that KD shows a better response in patients with recently increased seizure frequency than in patients with stable sporadic seizures [12]. However, our patient showed a great decrease in seizure frequency with mild cognitive improvement despite the initiation of KD in the chronic phase. Even in patients with chronic epilepsy resistant to multiple AEDs, KD should be fully considered to reduce seizure frequency and prevent further cognitive decline.

The appropriate duration of KD in refractory epilepsies, including FIRES, is controversial [8]. The antiepileptic effect can persist in some children with refractory epilepsies, including FIRES, after its discontinuation [5,13]. In addition, stopping KD may lead to increased seizure frequency and recurrent SE in some adult patients [10]. We consider that the following points are important to successfully continue KD: the application of more liberal regimens, multidisciplinary teams with expertise in pediatric and adult neurology, epileptology, nutritional science, and cooperation of the family offering KD at home such as in our case.

## Conclusions

We reported that a young adult patient with FIRES showed a significant decrease in seizure frequency due to long-term KD despite initiation in the chronic stage, while his cognitive function incompletely improved. Although early initiation of KD in FIRES is desirable to optimize both seizure control and cognitive outcome, we consider that KD can be applied for adult patients with chronic refractory epilepsy as well.
